# The Development of Global Women’s Rights and Improvements in Reproductive Health Intervention Access of Females with Different Socio-Economic Status

**DOI:** 10.3390/ijerph16234783

**Published:** 2019-11-28

**Authors:** Bocong Yuan, Jiannan Li, Zhaoguo Wang

**Affiliations:** 1 Center for Tourism Planning Development and Research, School of Tourism Management, Sun Yat-sen University, Guangzhou 510275, China; yuanbc@mail.sysu.edu.cn; 2 International School of Business & Finance, Sun Yat-sen University, Guangzhou 510275, China; 3 School of Economics and Management, Shenyang Agricultural University, Shenyang 110866, China

**Keywords:** health equity, reproductive health intervention, contraceptive access, women’s rights

## Abstract

Female’s access to reproductive health intervention has experienced dramatic change with the development of women’s rights across the world. However, the influence of the development of global women’s rights on reproductive health intervention access differs by place of residence and by the socio-economic characteristics of educational attainment and income levels. As a response to it, this study investigates the influence of the development of global women’s rights on contraceptive intervention access of females from different places of residence (rural/urban areas), with different educational attainment and income levels. Using multi-source data from World Health Organization (WHO), Inter-Parliament Union (IPU), International Labor Organization (ILO), and United Nations Educational, Scientific and Cultural Organization (UNESCO), empirical results show that the development of women’s rights generally improves female’s contraceptive intervention access around the world, and especially benefits females in rural areas, with a lower educational level, and in the medium or low-income stratum. The development of global women’s rights thus contributes to the social equity of healthcare access for females.

## 1. Introduction

The issue of female’s access to reproductive health intervention is, at the very beginning, beyond a pure medical problem and mixed with ethical, juridical, religious, and human rights controversies [1,2,3,4,5]. Female’s access to reproductive health intervention has long been excluded outside the priority of governmental, legislative, and juridical practice [1,2]. Many humanitarian areas composed mostly of the most vulnerable countries in Africa, the least developed countries, and the landlocked developing countries, are featured by the neglect of female’s reproductive health intervention needs and rights [6]. Despite a great deal of effort being devoted, gaps exist in accessing reproductive health intervention services, such as the access to emergency contraception in these areas [6]. Moreover, the legal and policy barrier impeding female’s access to reproductive health intervention has widely existed in the least developed countries, the low- and middle-income countries, and the small island developing states [6]. What’s worse, in some of these countries and regions, a political landscape with growing opposition towards reproductive health and reproductive rights is threatening the results achieved [6]. Even in a developed country like the United States, the juridical discussion and public concern of female’s reproductive health intervention rights have been shown to wax and wane since 1960. For example, the Griswold v. Connecticut (1965) makes an advance in this process. In this case, the Supreme Court ruled that the Constitution protected a right to privacy, whereby it invalidated the state law of Connecticut that prohibited contraceptive access [7,8]. Even so, the female autonomy to terminate her pregnancy was not legally recognized as a kind of right to privacy until several years later in the Roe v. Wade (1973) [9,10]. Nonetheless, such congressional and juridical confrontations have never stopped after that, and many efforts and subsequent measures are stimulated by various parties to constrain the implementation of this female right in reality [1,2,9].

At the present time, there is yet no place in the world endowing females with full freedom of reproductive health intervention, and there has long existed the social control over females, such as the censure of non-marital childbearing and the widespread prejudice against females who access reproductive health intervention [11,12]. Even though the universal human rights declarations admit that couples have the right of family planning, such right has rarely been accepted as the right of women as individuals [13,14].

During the past few decades, the most important driving force to continuously reshape female’s access to reproductive health intervention may not solely come from the political appeal or social movement. In reality, the development of social economy irreversibly improves the female status. The constantly rising female literacy and growing female participation in labor markets involve more and more women in wage-paid jobs [15,16,17]. The increase in female parliamentary representation and in female employers or supervisors can also partially reflect the development of women’s rights [18,19]. Several signs are observed that the development of women’s rights makes females start to be entitled with their reproductive autonomy in the timing and number of childbearing to some extent. For example, the countries with the higher female labor force participation rate are found with the late age of first reproduction and fewer children per household [20,21]. Besides, some countries with a higher female literacy rate, higher female school enrollment rate, and higher female educational level are found associated with a lower fertility rate on average. And this fact is partially attributed to the improvement in female’s consciousness of expanding career opportunities, of self-entitlement and autonomy, and of reluctance to spend more valuable time on childbearing [22,23,24].

However, the concern about the effect of women’s rights development on reproductive health intervention access still stays at the speculation stage with somewhat related signs/observations. This study thus intends to provide the empirical evidence for this concern. Through analyzing the national-level longitudinal data (during 2000–2015), this study can reveal the scientific findings and help avoid the fuzzy speculation. Moreover, the effect of women’s rights development on reproductive health intervention access may be discrepant for females from different residence places, with different educational attainment and income levels. This study thus further depicts the disparities in such effect between urban and rural females, well and less educated females, and wealthy and poorer females around the world, and in doing so gives findings of the significance in practice for national governments. Finally, the issue of female’s access to reproduction health intervention is a reflection of social equity in healthcare. Due to females living in rural areas, with a lower education and income level lacking abundant social-economic resources, improvement of their reproductive health intervention access may depend more on the top-down implementation of women’s rights protection. Thus, women’s rights development may serve as a powerful force to improve social equity in healthcare. As such, the investigation of disparity in the effect of women’s rights development on reproductive health intervention access between different female groups can, to some extent, bring policy makers some insights into the promotion of social equity in healthcare.

## 2. Materials and Methods

### 2.1. Data Source and Description

The data used in this study come from several sources including the reproductive health intervention section of Health Equity Monitor (HEM) published by the World Health Organization (WHO), Inter-Parliament Union (IPU), International Labor Organization (ILOSTAT database), and United Nations Educational, Scientific and Cultural Organization (UNESCO) Institute for Statistics. All of these datasets involve both low- and middle-income countries and high-income countries, and cover a time span of 15 years (during 2000–2015). The data are matched according to “country–year”. Given the missing values, the number of years might not be the same for different countries. Thus, the unbalanced longitudinal data are used in the regression analysis. Only the country–year observations where all the variables have no missing values can be regarded valid in the actual regression analysis. The list of countries that meet the condition of all the variables having no missing values in at least one year is provided in Appendix A.

The data of reproductive health intervention access categorized by the residence place (rural/urban), the education levels (none/primary/secondary or above), and the income levels (Q1, poorest–Q5, richest) are processed and published by the WHO. Women aged between 15 and 49, married or cohabitating, and currently using or whose sexual partners are using at least one modern method of contraception are surveyed by the WHO.

### 2.2. Variables

The dependent variables used in this study are “contraceptive access–modern method” and “contraceptive access–modern and traditional method”. The modern methods of contraception include the following ways: female and male sterilization, oral hormonal pills, the intra-uterine device, the male condom, injectables, the implant, vaginal barrier methods, the female condom, and emergency contraception. It is reported that traditional methods (usually referring to those except for modern ones) are prevalent in many underdeveloped countries or low-resource areas. For example, herbal contraception is popular in some Latin American countries [25], while periodic abstinence and folkloric ways (amulets, beads, etc.) are found popular in some sub-Saharan African and South Asian countries [26,27]. The United Nations report shows that the utilization rate of traditional methods can be high (5.6% for Asia, 6.1% for Latin America and the Caribbean, and 12.0% for Central Africa) [27,28]. The omission of those traditional methods usage can underestimate the access in underdeveloped countries. Considering traditional methods can vary across different countries and potential misinterpretation needs to be avoided, the WHO does not provide the precise definition of traditional methods, and states that the traditional methods refer to the other methods except for the modern ones.

Four variables that reflect the development of women’s rights are used as independent variables in this study, which include the percentage of female parliamentary seats in a country, the percentage of female employers to the whole female employment, the primary-school aged net enrollment rate for females, and the employment to population ratio for females (15 years-old and above). More details are shown in Table 1. There are several reasons why these four independent variables are included in the study. First, these variables provide the acceptable proxies for many facets of women’s rights for a country (i.e., political rights, educational rights, economic rights), and are suggested by many previous studies [29,30,31]. Second, political rights, educational rights, and economic rights get the most attention in previous studies regarding the relations between women’s rights and reproductive health [32,33]. Third, these variables/indexes are among the main concerns of many international organizations, and are surveyed and published by these organizations. The resulting open-source data of these variables make it feasible for other researchers to conduct the replication and verifiability of research findings.

### 2.3. Method

This study evaluates the effect of global women’s rights development on reproductive health intervention access. Through comparing such effect between rural and urban areas, between female population with different education levels (none, primary, secondary, and above), and different income levels (from the poorest to the richest), this study could reveal the situation of equity in reproductive health intervention access around the world.

The fixed effect model is used in this study to capture the unobservable heterogeneity of different countries. The fixed effect model is effective in coping with the situation where there exists the intrinsic difficulty in controlling every potential determinant in the regression analysis. In the context of this study, there can also be other factors affecting female reproductive health intervention access of a specific country (e.g., religion), however, such data on the country level are rarely available. In this case, the use of common regression analysis (i.e., ordinary least square, OLS) might incur biased estimation for omission of relevant variables. In contrast, the fixed effect model can successfully control the unobservable heterogeneity by estimating the country-specific intercept that varies in different countries [34]. All the variables are taken natural log (Ln), and the estimated coefficients reflect the elasticity (i.e., the percentage change of the dependent variable corresponding to the percentage change of independent variables) [35]. The regression of this model is shown as below:

Ln[Contraceptive prevalence]_it_ = β_0 i_ + β_1_Ln[Parliament]_it_ + β_2_ Ln[Employer]_it_ + β_3_ Ln[Enrollment]_it_ + β_4_ Ln[Employment-population ratio]_it_ + ε _it_, (1)

where i indicates the i-th country, j indicates the j-th year, and β_0_ i indicates a country-specific intercept that varies in different countries.

## 3. Results

Table 2 shows the descriptive statistical analysis of all the variables during 2000–2015 (including mean values, standard deviation, the range from the min to the max, and the number of non-missing observations). The detailed descriptive statistical analysis of the dependent variable categorized by the residence place (rural/urban), the education levels (none/primary/secondary or above), and the income levels (Q1, poorest–Q5, richest) is also provided in it.

Table 3 shows the effects of women’s rights development on contraceptive access of females from different residence places. The regression coefficients show that the development of women’s rights can positively predict contraceptive access of both urban and rural females, besides, such effect for rural females is statistically significant and greater than that for urban counterparts. Meanwhile, these regression results are shown robust, no matter if the “modern method” or “modern and traditional method” is used to measure contraceptive access.

Table 4 shows the effects of women’s rights development on contraceptive access of females with different educational attainment levels. The regression results show that the development of women’s rights has a positive effect on contraceptive access of females with different educational attainment levels. Besides, such effect is shown greater for females with none and primary education level than for females with secondary education level. And these results are shown robust no matter if the “modern method” or “modern and traditional method” is used to measure contraceptive access.

Table 5 shows the effects of women’s rights development on contraceptive access of females with different income levels. The regression results show that the development of women’s rights generally has a positive effect on contraceptive access of females with different income levels. In addition, such effect, in most cases, is greater for females in the lower-to-medium stratum income level (Q1, Q2, and Q3) than for females in the higher stratum income level (Q4 and Q5). And regression results above are shown robust no matter if the “modern method” or “modern and traditional method” is used to measure contraceptive access.

**Figure A1:**
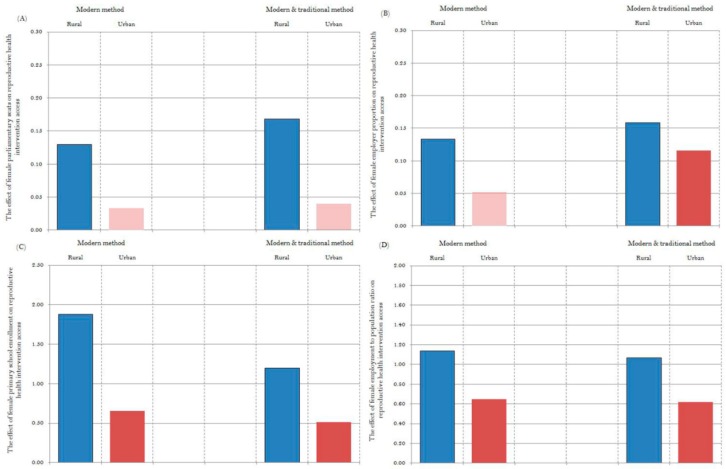
The effect of female parliamentary seats (**A**), female employer proportion (**B**), female primary school enrollment (**C**), and female employment to population ratio (**D**) on reproductive health intervention access (residence place difference). Notes: The translucent column indicates that the effect is statistically insignificant; the height of the columns indicates the magnitude of effect.

**Figure A2:**
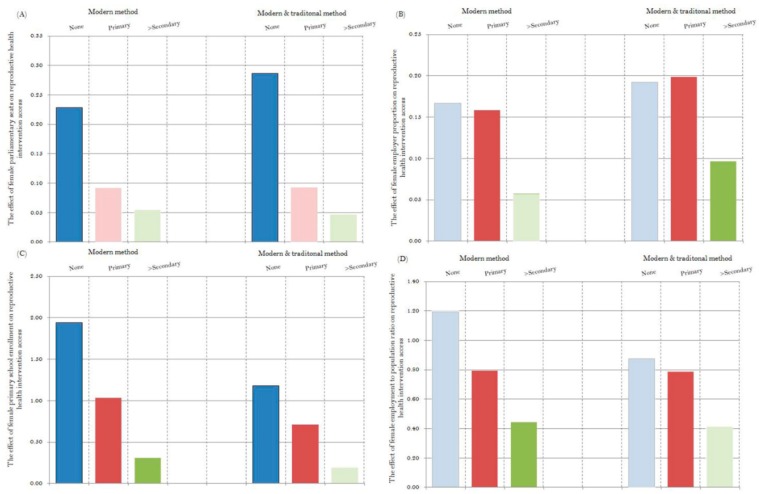
The effect of female parliamentary seats (**A**), female employer proportion (**B**), female primary school enrollment (**C**), and female employment to population ratio (**D**) on reproductive health intervention access (education difference). Notes: The translucent column indicates that the effect is statistically insignificant; the height of the columns indicates the magnitude of effect.

**Figure A3:**
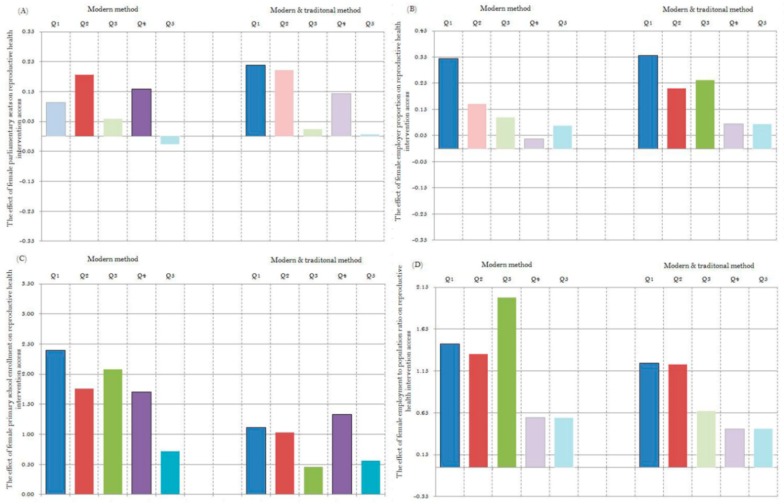
The effect of female parliamentary seats (**A**), female employer proportion (**B**), female primary school enrollment (**C**), and female employment to population ratio (**D**) on reproductive health intervention access (income difference). Notes: The translucent column indicates that the effect is statistically insignificant; the height of the columns indicates the magnitude of effect.

Taken above results together, it can be concluded that the development of women’s rights could positively affect contraceptive access of females, and such effect is discrepant for different female groups. Those living in rural areas, with a lower educational level, and in the lower-to-medium stratum income level, could benefit more from the development of women’s rights. Their contraceptive access is shown to increase more greatly with the improvement of women’s rights. The comparison of the above effects is more clearly illustrated in Figure A1, Figure A2 and Figure A3 (see details in Appendix B).

## 4. Discussion and Conclusions

This study provides an empirical investigation on how the development of women’s rights affects reproductive health intervention access of females worldwide. This study finds that the access of rural females to reproductive health interventions can benefit more from the development of women’s rights than their urban counterparts. Generally, the higher economic status in the family may entitle urban females to be able to access contraception on their own volition [36,37]. Moreover, urban females are more likely to access greater choices about reproductive health services they need, given the urban–rural disparity in healthcare throughout the world [38]. Conversely, for rural females, lower economic status may lead them to depend more on outside force, such as the government-led reform, to improve their reproductive health intervention access. Thus, compared to urban females worldwide, the improvement of reproductive health intervention access for rural females could be more profoundly influenced by the overall progress of women’s rights.

Moreover, lower levels of education can cause females to be unconscious of their own rights and entitlements [38,39]. Their values and visions are deeply shaped by the traditional gender norms established by culture, clan, the older generation, and men’s expectations [40,41]. Despite legally proclaimed equal rights between females and males, many biased normative forces continue to operate to hold undereducated females back [42,43]. They bind females in the shackles of traditional social roles and often deprive them of the autonomy of reproductive choice. Conversely, the nurture of education can help change traditional negative attitudes towards females. Well-educated females have new values and visions of their own rights and entitlements. They are motivated to be free from a lifetime of childbearing and, instead, to decide for themselves the timing of childbearing, the number and spacing of children, and the choice of contraceptive method [44,45]. As such, the top-down implementation of women’s rights protection is necessarily the much more effective solution for undereducated females to obtain greater reproduction choice, whereas their well-educated counterparts have self-awareness to acquire and practice their rights of reproduction.

Further, the income level of females can largely influence the amount and quality of healthcare resources they can access. Females in the high stratum income level generally have strong ability to pay for the high quality and wide range of healthcare services [46]. However, females of poor economic status have to save money in low-priority areas to cover necessary expenditure on children, elderly, and basic living. Their own needs of reproduction are always secondary to feeding their family [47]. By minimizing some expense in contraceptive methods, they actively reject the access to reproductive health intervention to some degree. Therefore, compared with females in the high stratum income level, the improvement of reproductive health intervention for females of poor economic status could depend much more on the top-down implementation of women’s rights protection.

Generally, rural areas suffer from the lower level of education and income. It is thus more likely that these characteristics are interwoven with each other in rural females. Hence, it is most important to increase school enrollment for rural females and spare no efforts to equalize the educational opportunities for them around the country. It is also important that interventions help rural females to be aware of and to understand the various modes of gender discrimination that have been embedded in the social phenomena of their daily lives. The optimal status of female education is when the whole society becomes conscious of gender bias and willing to share opportunities and other valuable resources. Rural females who are fully aware of their own reproduction rights and entitlements are motivated to advocate reproductive health empowerment of females. Built on this basis, the top-down reform could more effectively lead to genuine advances in women’s rights of reproductive health intervention. Moreover, employment training for rural females is necessary. This practice can help enhance their employability and economic independence, which is the foundation to protect women’s rights. Finally, governments can create a plan to set up a series of policies, from which rural females can access the same services or goods of reproduction health intervention with less money. This measure aims to heal the sharp divide in the access to reproduction health interventions between rural and urban females, or between poorer and wealthy females. It must be recognized that top-down approaches will not always be successful due to numerous reasons, such as a lack of government commitment, ineffective implementation, the complex web of traditions and customs about marriage, family, spousal relations, and gender dynamics behind the utilization of contraceptives [48]. These factors thus need to be considered to pave the way for the successful implementation of top-down approaches.

Still, we must admit that the choice of indicators of women’s rights is not unique, and the availability of data can constrain our effort to make further exploration of other relevant or more meaningful indicators. For example, we have a few reasons why we use the employment to population ratio of females to proxy women’s economic rights to some extent. First, females who participate in labor market and earn wages could have higher economic status in their family than their counterparts living on their husbands’ earning. And thus, they can place more weight of family wealth on their own reproductive health. This situation could exist in both developed and developing countries. Second, a society with higher labor participation of females may imply that females in this society can pursue their own careers rather than bind their life to childbearing. And thus, females in such society could have more freedom to reproductive health interventions. Third, a higher level of female labor participation might at least reflect the more employment opportunities open to females and the less employment discrimination blocking females outside the labor market. Even though those reasons seem sound, we must admit that this indicator is not free of flaw. For example, females in developing countries participating in the labor market might just work out of necessity. Higher employment to population ratio of females might imply poverty or lower development level of that area. In contrary, higher employment to population ratio of females in developed countries might seem a more appropriate proxy of women’s economic rights. The above discussion might explain, to some extent, why we find the influence of employment to population ratio of females on contraceptive access of females with no education as insignificant.

## Figures and Tables

**Table 1:** Description of independent variables.

Independent Variables	Abbreviation	Definition	Data Source
Ln [Proportion of seats held by women in national parliaments (in percentage)]	Ln[Parliament]	Women in parliaments are the percentage of parliamentary seats in a single or lower chamber held by women.	Inter-Parliamentary Union (IPU)
Ln [Employers, female (in percentage, of female employment) (modeled ILO estimate)]	Ln[Employer]	Employers indicate workers who work on their own account or with one or a few partners, hold the type of jobs defined as “self-employment jobs” (i.e., jobs where the remuneration is directly dependent upon the profits derived from the goods and services produced), and in this capacity, have engaged, on a continuous basis, one or more persons to work for them as employee(s).	International Labor Organization, ILOSTAT database.
Ln [Adjusted net enrollment rate, primary, female (in percentage, of primary school age children)]	Ln[Enrollment]	Adjusted net enrollment is the number of pupils of the school-age group for primary education, enrolled either in primary or secondary education, expressed as a percentage of the total population in that age group.	UNESCO Institute for Statistics
Ln [Employment to population ratio, 15+, female (in percentage) (modeled ILO estimate)]	Ln[Employment population ratio]	Employment is defined as persons of working age who, during a short reference period, were engaged in any activity to produce goods or provide services for pay or profit, whether at work during the reference period (i.e., who worked in a job for at least one hour) or not at work due to temporary absence from a job, or to working-time arrangements. Ages 15 and older are generally considered the working-age population.	International Labor Organization, ILOSTAT database.

**Table 2:** The overview of variables (2000–2015).

Variables	Mean	S.D.	Range	Non-Missing Observation
Dependent variables				
Residence place difference				
Ln [Contraceptive access]—Modern method—Rural	3.0534	1.0780	[−1.6094, 4.3618]	258
Ln [Contraceptive access]—Modern method—Urban	3.5075	0.6218	[0.8755, 4.3399]	259
Ln [Contraceptive access]—Modern & traditional method—Rural	3.4347	0.8416	[0.8329, 4.4018]	258
Ln [Contraceptive access]—Modern & traditional method—Urban	3.7653	0.5157	[1.5892, 4.4006]	259
Educational difference				
Ln [Contraceptive access]—Modern method—None	2.7878	1.1187	[−0.6931, 4.3503]	224
Ln [Contraceptive access]—Modern method—Primary	3.2479	0.8070	[0.7419, 4.3994]	239
Ln [Contraceptive access]—Modern method—Secondary or above	3.5778	0.5210	[1.8406, 4.3307]	258
Ln [Contraceptive access]—Modern & traditional method—None	3.1440	0.8912	[0.9933, 4.3770]	224
Ln [Contraceptive access]—Modern & traditional method—Primary	3.5737	0.6203	[1.8083, 4.4164]	240
Ln [Contraceptive access]—Modern & traditional method—Secondary or above	3.8431	0.4112	[2.3026, 4.4092]	258
Income difference				
Ln [Contraceptive access]—Modern method—Q1[poorest]	2.7869	1.2658	[−2.3026, 4.3944]	254
Ln [Contraceptive access]—Modern method—Q2	2.9870	1.1695	[−1.6094, 4.3669]	255
Ln [Contraceptive access]—Modern method—Q3	3.1699	1.0546	[−1.6094, 6.5958]	257
Ln [Contraceptive access]—Modern method—Q4	3.3510	0.8402	[−0.9163, 4.3748]	256
Ln [Contraceptive access]—Modern method—Q5[richest]	3.5763	0.5683	[1.2801, 4.2541]	254
Ln [Contraceptive access]—Modern & traditional method—Q1[poorest]	3.2167	0.9908	[0.2624, 4.4128]	255
Ln [Contraceptive access]—Modern & traditional method—Q2	3.3744	0.9158	[0, 4.4031]	255
Ln [Contraceptive access]—Modern & traditional method—Q3	3.5133	0.7952	[0.9163, 4.4320]	255
Ln [Contraceptive access]—Modern & traditional method—Q4	3.6462	0.6695	[1.2528, 4.4308]	255
Ln [Contraceptive access]—Modern & traditional method—Q5[richest]	3.8286	0.4649	[1.9459, 4.4140]	255
Independent variables				
Ln [Parliament]	2.6188	0.7679	[−1.2040, 4.1558]	2757
Ln [Employer]	0.1502	1.0148	[−3.6119, 2.3817]	2848
Ln [Enrollment]	4.4581	0.2275	[3.0762, 4.6052]	1714
Ln [Employment population ratio]	3.7626	0.4357	[1.5007, 4.4545]	2848

Notes: All variables in the regression are in the form of natural log.

**Table 3:** Development of women’s rights and reproductive health intervention access (residence place difference).

Variables	Dependent Variable: Ln [Contraceptive Access]			
	Modern Method		Modern & Traditional Method	
	Rural	Urban	Rural	Urban
Ln [Parliament]	0.1293 * [0.0726]	0.0331 [0.0464]	0.1680 ** [0.0791]	0.0404 [0.0530]
Ln [Employer]	0.1331 * [0.0774]	0.0509 [0.0495]	0.1584 * [0.0843]	0.1159 ** [0.0564]
Ln [Enrollment]	1.8766 *** [0.1773]	0.6545 *** [0.1134]	1.1951 *** [0.1931]	0.5110 *** [0.1294]
Ln [Employment population ratio]	1.1342 ** [0.4514]	0.6487 ** [0.2286]	1.0691 ** [0.4915]	0.6188 * [0.3293]
Intercept [the average of unobserved heterogeneity]	−9.8037 *** [1.9162]	−1.8757 *** [1.2253]	−6.3456 *** [2.0866]	−0.9196 [1.3978]
Number of observations	139	140	139	140
Number of countries	71	72	71	72
R^2^ (within)	0.7160	0.4375	0.5395	0.3282
R^2^ (between)	0.1062	0.0151	0.0581	0.0254
R^2^ (overall)	0.2089	0.0471	0.1586	0.0885
σ_u_	0.9657	0.6190	0.9023	0.5443
σ_e_	0.1707	0.1092	0.1859	0.1245
ρ	0.9697	0.9698	0.9593	0.9503
F-statistics [*p*-value]	40.33 [0.0000]	12.44 [0.0000]	18.75 [0.0000]	7.82 [0.0000]

Notes: All variables in the regressions are in the form of natural log, and thus the estimated coefficients reflect the elasticity (i.e., the percentage change of the dependent variable corresponding to the percentage change of the independent variables). σ_u_ = individual variance. σ_e_ = random disturbance variance. ρ = fraction of individual variance. * *p* < 0.10, ** *p* < 0.05, *** *p* < 0.01.

**Table 4:** Development of women’s rights and reproductive health intervention access (Education difference).

Variables	Dependent Variable: Ln [Contraceptive Access]					
	Modern Method			Modern & Traditional Method		
	None	Primary	Secondary or above	None	Primary	Secondary or above
Ln [Parliament]	0.2284 * [0.1212]	0.0921 [0.0566]	0.0541 [0.0405]	0.2866 ** [0.1229]	0.0923 [0.0683]	0.0467 [0.0482]
Ln [Employer]	0.1665 [0.1332]	0.1586 ** [0.0595]	0.0575 [0.0432]	0.1920 [0.1350]	0.1986 *** [0.0718]	0.0965 * [0.0514]
Ln [Enrollment]	1.9389 *** [0.2814]	1.0337 *** [0.1322]	0.3107 *** [0.0992]	1.1802 ** [0.2853]	0.7138 *** [0.1596]	0.1913 [0.1179]
Ln [Employment population ratio]	1.1934 [0.7576]	0.7943 ** [0.3542]	0.4436 * [0.2525]	0.8750 [0.7681]	0.7863 * [0.4274]	0.4118 [0.3001]
Intercept [the average of unobserved heterogeneity]	−10.8523 *** [3.1951]	−4.4834 *** [1.4896]	0.4441 [1.0719]	−6.1576 * [3.2395]	−2.7812 [1.7979]	1.3371 [1.2739]
Number of observations	121	129	140	121	129	140
Number of countries	62	65	72	62	65	72
R^2^ (within)	0.5871	0.6337	0.2814	0.4416	0.4448	0.1778
R^2^ (between)	0.0266	0.0127	0.0096	0.0361	0.0245	0.0203
R^2^ (overall)	0.1202	0.0697	0.0330	0.1473	0.1155	0.0772
σ_u_	1.1819	0.8455	0.5125	0.9640	0.7050	0.4247
σ_e_	0.2685	0.1265	0.0954	0.2723	0.1526	0.1134
ρ	0.9509	0.9781	0.9665	0.9261	0.9552	0.9334
F-statistics [*p*-value]	19.55 [0.0000]	25.95 [0.0000]	6.26 [0.0003]	10.87 [0.0000]	12.02 [0.0000]	3.46 [0.0127]

Notes: All variables in the regressions are in the form of natural log, and thus the estimated coefficients reflect the elasticity (i.e., the percentage change of the dependent variable corresponding to the percentage change of independent variables). σ_u_ = individual variance. σ_e_ = random disturbance variance. ρ = fraction of individual variance. * *p* < 0.10, ** *p* < 0.05, *** *p* < 0.01.

**Table 5:** Development of women’s rights and reproduction health intervention access (income difference).

Variables	Dependent Variable: Ln [Contraceptive Access]									
	Modern Method					Modern & Traditional Method				
	Q1 [Poorest]	Q2	Q3	Q4	Q5 [Richest]	Q1 [Poorest]	Q2	Q3	Q4	Q5 [Richest]
Ln [Parliament]	0.1134 [0.1079]	0.2067 * [0.1107]	0.0595 [0.1371]	0.1578 ** [0.0669]	−0.0262 [0.0696]	0.2386 ** [0.1174]	0.2219 ** [0.0986]	0.0239 [0.0926]	0.1435 ** [0.0677]	0.0070 [0.0375]
Ln [Employer]	0.3446 *** [0.1205]	0.1715 [0.1149]	0.1184 [0.1531]	0.0367 [0.0747]	0.0866 [0.0777]	0.3554 *** [0.1305]	0.2305 ** [0.1096]	0.2619 ** [0.1030]	0.0942 [0.0754]	0.0937 [0.0595]
Ln [Enrollment]	2.3945 *** [0.2640]	1.7615 *** [0.2537]	2.0798 *** [0.3354]	1.7038 *** [0.1635]	0.7197 *** [0.1702]	1.1133 *** [0.2876]	1.0320 *** [0.2415]	0.4575 ** [0.2268]	1.3256 *** [0.1660]	0.5626 *** [0.1304]
Ln [Employment population ratio]	1.4749 ** [0.6707]	1.3534 ** [0.6387]	2.0288 ** [0.8541]	0.5962 [0.4180]	0.5879 [0.4326]	1.2464 * [0.6959]	1.2304 ** [0.5844]	0.6680 [0.5519]	0.4593 [0.4037]	0.4611 [0.3314]
Intercept [the average of unobserved heterogeneity]	−13.6633 *** [2.8564]	−10.4350 *** [2.7223]	−13.8455 *** [3.6350]	−6.7807 ** [1.7768]	−1.7197 [1.8422]	−7.0850 ** [2.9799]	−6.4335 ** [2.5025]	−1.0773 [2.3591]	−4.2990 ** [1.7257]	−0.4005 [1.4114]
Number of observations	136	133	136	135	136	141	141	140	139	136
Number of countries	69	68	69	69	69	71	71	71	70	69
R^2^ (within)	0.6544	0.5800	0.4587	0.7162	0.2556	0.4173	0.4394	0.1842	0.6027	0.2947
R^2^ (between)	0.0901	0.0470	0.0132	0.1199	0.0208	0.0349	0.0281	0.0307	0.1536	0.0468
R^2^ (overall)	0.1999	0.1427	0.0712	0.2683	0.0717	0.1248	0.1162	0.1209	0.3324	0.1416
σ_u_	1.2194	1.1436	1.3348	0.6898	0.5532	1.1247	1.0263	0.7880	0.5841	0.4480
σ_e_	0.2539	0.2408	0.3225	0.1572	0.1638	0.2769	0.2326	0.2184	0.1598	0.1255
ρ	0.9584	0.9575	0.9448	0.9506	0.9194	0.9428	0.9512	0.9287	0.9304	0.9273
F-statistics [*p*-value]	29.82 [0.0000]	21.06 [0.0000]	13.35 [0.0000]	39.12 [0.0000]	5.41 [0.0008]	11.82 [0.0000]	12.93 [0.0000]	3.67 [0.0093]	24.65 [0.0000]	6.58 [0.0002]

Notes: All variables in the regressions are in the form of natural log, and thus the estimated coefficients reflect the elasticity (i.e., the percentage change of the dependent variable corresponding to the percentage change of independent variables). σ_u_ = individual variance. σ_e_ = random disturbance variance. ρ = fraction of individual variance. * *p* < 0.10, ** *p* < 0.05, *** *p* < 0.01.

## Appendix A

**Table A1:** List of countries.

Albania	Djibouti	Lesotho	Peru
Argentina	Egypt, Arab Rep.	Madagascar	Philippines
Armenia	El Salvador	Malawi	Moldova
Azerbaijan	Eswatini	Maldives	Sao Tome and Principe
Belarus	Ethiopia	Mali	Senegal
Belize	Gambia, The	Mauritania	Serbia
Benin	Georgia	Mexico	Sierra Leone
Bhutan	Ghana	Mongolia	Sudan
Bolivia	Guatemala	Morocco	Suriname
Burkina Faso	Guinea	Mozambique	Syrian Arab Republic
Burundi	Guyana	Namibia	Tajikistan
Cambodia	Honduras	Nepal	North Macedonia
Cameroon	Indonesia	Nicaragua	Timor-Leste
Central African Republic	Jordan	Niger	Uganda
Colombia	Kazakhstan	Nigeria	Ukraine
Congo, Rep.	Kenya	Pakistan	Tanzania
Costa Rica	Kyrgyz Republic	Panama	Yemen, Rep.
Cuba	Lao PDR	Paraguay	Zambia

Notes: The list of countries corresponds to the regression of Table 3 (the 2nd column), and has 72 countries in total. Since the number of non-missing observations varies for each regression and the country list might have slight difference for each regression, it might seem redundant to report several country lists corresponding to every regression. We choose to report the country list corresponding to the regression of Table 3 (the 2nd column), as this list covers the most countries (all other regressions cover no more than 72 countries).

## Appendix B. The Illustration of Regression Results

Figure A1, Figure A2 and Figure A3, in a visual-friendly way, depict the disparities in the effect of women’s rights development on contraceptive access between urban and rural females, well- and less-educated females, and wealthy and poorer females around the world.

In Figure A1, the heights of the columns representing rural females are obviously greater than that for urban females, and some columns for urban females are translucent. These results indicate that for rural females, the effect of women’s rights development on contraceptive access is significant and the magnitude of such effect is greater than that for urban counterparts.

In Figure A2, the heights of the columns for females with none and primary education level are greater than that for females with secondary education level, and most of the columns for females with secondary education level are translucent. These results demonstrate that the effect of women’s rights development on contraceptive access for females with lower level of education is more significant and greater than that for counterparts with higher level of education.

Figure A3 shows that most of the columns for females in the higher stratum income level (Q4 and Q5) are translucent, and the heights of the columns for females in the lower-to-medium stratum income level (Q1, Q2, and Q3) are, in most cases, greater than that for females in the higher stratum income level (Q4 and Q5). These results indicate that the effect of women’s rights development on contraceptive access for females in the lower-to-medium stratum income level is more significant and greater than that for counterparts in the higher stratum income level.

## References

[1] Cook R.J. (1993). International human rights and women’s reproductive health. Stud. Fam. Plan..

[2] Cook R.J. (1994). Human rights and reproductive self-determination. Am. Univ. Law Rev..

[3] Di Mauro D., Joffe C. (2007). The religious right and the reshaping of sexual policy: An examination of reproductive rights and sexuality education. Sex. Res. Soc. Policy.

[4] Freedman L.P., Isaacs S.L. (1993). Human rights and reproductive choice. Stud. Fam. Plan..

[5] Schenker J.G., Eisenberg V.H. (1997). Ethical issues relating to reproduction control and women’s health. Int. J. Gynecol. Obstet..

[6] United Nations Population Fund UNFPA Statistic Plan, 2018–2021. https://www.unfpa.org/sites/default/files/pub-pdf/18-044_UNFPA-SP2018-EN_2018-03-12-1244_0.pdf.

[7] Bailey M.J. (2010). Momma’s got the pill: How Anthony Comstock and Griswold v. Connecticut shaped US childbearing. Am. Econ. Rev..

[8] Helscher D. (1994). Griswold v. Connecticut and the unenumerated right of privacy. North. Ill. Univ. Law Rev..

[9] Ginsburg R.B. (1984). Some thoughts on autonomy and equality in relation to Roe v. Wade. N. C. Law Rev..

[10] Davis P.C. (1993). Neglected stories and the lawfulness of Roe v. Wade. Harv. Civ. Right-Civ. Lib. Law Rev..

[11] Bader V., Kelly P.J., Cheng A.L., Witt J. (2014). The role of previous contraception education and moral judgment in contraceptive use. J. Midwifery Women’s Health.

[12] Moen E. (1981). Women’s rights and reproductive freedom. Hum. Rights Q..

[13] Hewson B. (2001). Reproductive autonomy and the ethics of abortion. J. Med. Ethics.

[14] Cameron L.A., Malcolm Dowling J., Worswick C. (2001). Education and labor market participation of women in Asia: Evidence from five countries. Econ. Dev. Cult. Chang..

[15] Ince M. (2010). How the education affects female labor force? Empirical evidence from Turkey. Procedia-Soc. Behav. Sci..

[16] Mitra A., Singh P. (2006). Human capital attainment and female labor force participation—The Kerala puzzle. J. Econ. Issues.

[17] Halder N. (2004). Female representation in parliament: A case study from Bangladesh. N. Z. J. Asian Stud..

[18] Özbilgin M. (2009). Equality, Diversity and Inclusion at Work: Yesterday, Today and Tomorrow.

[19] Gustafsson S. (2001). Optimal age at motherhood. Theoretical and empirical considerations on postponement of maternity in Europe. J. Popul. Econ..

[20] Mishra V., Smyth R. (2010). Female labor force participation and total fertility rates in the OECD: New evidence from panel cointegration and Granger causality testing. J. Econ. Bus..

[21] Bbaale E., Mpuga P. (2011). Female education, contraceptive use, and fertility: Evidence from Uganda. Consilience.

[22] Drèze J., Murthi M. (2001). Fertility, education, and development: Evidence from India. Popul. Dev. Rev..

[23] Osili U.O., Long B.T. (2008). Does female schooling reduce fertility? Evidence from Nigeria. J. Dev. Econ..

[24] Wooldridge J.M. (2010). Econometric Analysis of Cross Section and Panel Data.

[25] Bull S.S. (1998). Contraception and culture: The use of yuyos in Paraguay. Health Care Women Int..

[26] Bankole A., Ezeh A.C. (1999). Unmet need for couples: An analytical framework and evaluation with DHS data. Popul. Res. Policy Rev..

[27] Rossier C., Corker J. (2017). Contemporary use of traditional contraception in sub-Saharan Africa. Popul. Dev. Rev..

[28] United Nations Population Division World Contraceptive Patterns 2013. http://www.un.org/en/development/desa/population/publications/family/contraceptive-wallchart-2013.shtml.

[29] Anyanwu J.C. (2016). Analysis of gender equality in youth employment in Africa. Afr. Dev. Rev..

[30] Maclure R., Denov M. (2009). Reconstruction versus transformation: Post-war education and the struggle for gender equity in Sierra Leone. Int. J. Educ. Dev..

[31] Paxton P., Hughes M.M., Painter M.A. (2010). Growth in women’s political representation: A longitudinal exploration of democracy, electoral system and gender quotas. Eur. J. Political Res..

[32] Al Riyami A., Afifi M., Mabry R.M. (2004). Women’s autonomy, education and employment in Oman and their influence on contraceptive use. Reprod. Health Matters.

[33] Yuan B., Li J. (2019). Women’s rights development and reproductive health interventions access worldwide. Popul. Health Manag..

[34] Srivastava N., Srivastava R. (2010). Women, work, and employment outcomes in rural India. Econ. Political Wkly..

[35] Gyimah S.O., Adjei J.K., Takyi B.K. (2012). Religion, contraception, and method choice of married women in Ghana. J. Relig. Health.

[36] Mahapatro S.R. (2012). Utilization of maternal and child health care services in India: Does women’s autonomy matter?. J. Fam. Welf..

[37] Li J., Shi L., Liang H., Ding G., Xu L. (2018). Urban-rural disparities in health care utilization among Chinese adults from 1993 to 2011. BMC Health Serv. Res..

[38] Chigbu C.O., Onyebuchi A.K., Onwudiwe E.N., Iwuji S.E. (2013). Denial of women’s rights to contraception in southeastern Nigeria. Int. J. Gynecol. Obstet..

[39] Noreen G., Khalid H. (2012). Gender empowerment through women’s higher education: Opportunities and possibilities. J. Res. Reflect. Educ..

[40] Berik G. (2018). Understanding the Gender System in Rural Turkey: Fieldwork Dilemmas of Conformity and Intervention. Feminist Dilemmas in Fieldwork.

[41] Liu J., Carpenter M. (2005). Trends and issues of women’s education in China. Clear. House J. Educ. Strateg. Issues Ideas.

[42] Ambreen M., Mohyuddin A. (2013). Gender biased parental attitudes towards education: A case study of village dasuha, district Faisalabad. Acad. Res. Int..

[43] Pearse R., Connell R. (2016). Gender norms and the economy: Insights from social research. Fem. Econ..

[44] Derose L.F., Wu L., Dodoo F.N.A. (2010). Inferring gender-power: Women’s schooling and relative spousal influence in childbearing in Ghana. Genus.

[45] Camporesi S. (2017). Bioethics and biopolitics: Presents and futures of reproduction. J. Bioethical Inq..

[46] Umar A.S., Kennedy C., Tawfik H. (2017). Female economic empowerment as a significant factor of social exclusion on the use of antenatal and natal services in Nigeria. MOJ Women’s Health.

[47] Grant U. (2005). Health and Poverty Linkages: Perspectives of the Chronically Poor.

[48] Steinfield L.A., Coleman C.A., Tuncay Zayer L., Ourahmoune N., Hein W. (2019). Power logics of consumers’ gendered (in) justices: Reading reproductive health interventions through the transformative gender justice framework. Consum. Mark. Cult..

